# Single-cell profiling of H3K4me1-H3K27me3 revealed bivalent regulation of abnormal neuronal development caused by prenatal e-cigarette vaporing

**DOI:** 10.1038/s42003-025-08683-8

**Published:** 2025-09-01

**Authors:** Zhong Chen, Lijuan Song, Wanqiu Chen, Charles Wang

**Affiliations:** 1 Center for Genomics, Loma Linda University School of Medicine, Loma Linda, CA USA; 2 Department of Basic Sciences, Loma Linda University School of Medicine, Loma Linda, CA USA

**Keywords:** Epigenetics and plasticity, Epigenomics

## Abstract

Histone H3K4me1 and H3K27me3 modifications play a crucial role in regulating neuronal development by maintaining the balance between active and inactive genes during neurogenesis. Prenatal exposure to electronic-cigarette (e-cig) aerosol has been shown to alter neuronal differentiation in a neuron type-specific manner. However, it remains unclear whether e-cig aerosol exposure affects gene expression by altering H3K4me1 and H3K27me3 modifications. Using single-nucleus joint profiling of  H3K4me1-H3K27me3 and transcriptome of neonatal rat prefrontal cortex, we demonstrate that e-cig aerosol exposure alters the H3K4me1-H3K27me3 methylation patterns in the promoters, *i.e*., the bivalency, of many cell type-specific genes, impacting gene expression levels, neuronal differentiation and functions. Additionally, the prenatal e-cig aerosol exposure impacts the expression of genes related to circadian entrainment, calcium signaling, protein kinase signaling transduction, and synaptic transmission. These results suggest that nicotine addiction may be epigenetically imprinted at a very early stage of brain development.

## Abstract (web summary)

Prenatal e-cigarette aerosol exposure influences the neuronal development of the neonatal rat brain by modifying the bivalency of H3K4me1 and H3K27me3 at the promoters of genes specific to neuronal regulation.

## Introduction

E-cigarettes, also known as vaping, contain nicotine and other harmful substances, including flavoring agents and additives, can easily cross the placental barrier and affect the developing fetal brain. These substances can have neurotoxic effects and interfere with critical processes such as synaptogenesis, neurotransmitter signaling, and neuronal connectivity, which are essential for proper brain development^1,2^. Studies have shown that maternal e-cig smoking during pregnancy is associated with various adverse effects on offspring brain development, resulting in long-term consequences such as cognitive impairments, behavioral changes, and an increased risk of neurodevelopmental disorders in children^3,4^. In previous study, we showed that prenatal e-cig aerosol exposure altered neuron differentiation by changing the chromatin accessibility at the promoter regions of genes involved in neuron development, specifically, it promoted excitatory neuron (E) and hampered inhibitory neuron (I) differentiation, leading to E/I ratio imbalance^5^. However, it is unknown whether another layer of epigenetic modification, namely histone methylation, may be a target of epigenetic regulation induced by prenatal e-cig aerosol exposure during the early rat brain development stages.

Histone methylation at lysine residues is relatively stable and considered potential marks for carrying the epigenetic information that is stable through cell divisions^6,7^, thus provides a potential mechanism for long-term regulation of gene expression that could maintain the effects of early nicotine exposure into adulthood^8^. Histone H3 lysine K4 (H3K4) methylation is usually associated with transcriptional activation, while histone H3 lysine 27(H3K27) methylation is typically associated with transcriptional repression and is best known for its role during cell-fate determination^9,10^.

Single-cell RNA sequencing (scRNA-seq) is a powerful tool which enables researchers to uncover the heterogeneity of cell populations in the brain, identify rare cell types, and key regulators critical for brain development. Methods have also been described to profile histone modifications at single cell level^11,12^. However, histone modifications vary greatly in different cells, and directly correlating cell type-specific gene expression with histone modifications was challenging. Recently, a scalable strategy called Paired-Tag (parallel analysis of individual cells for RNA expression and DNA from targeted tagmentation by sequencing) was introduced for joint analysis of transcriptome and histone modification from the same single cells^13^. Taking advantage of the Paired-Tag technology, we simultaneously analyzed transcriptome along with H3K4me1 and H3K27me3 modifications from the same single nuclei isolated from postnatal day 7 rat prefrontal cortex following prenatal e-cig aerosol or control air exposure. We aimed to investigate the effects of prenatal e-cig smoking on histone marks H3K4m1 and H3K27me3 as well as their collaborative roles in regulating gene expression in specific cell types. We chose prefrontal cortex (PFC) as our region of interest because PFC is involved in higher-order brain functions such as cognition, learning, memory, reward and addiction^14^. PFC is vulnerable to a variety of stimuli, such as nicotine^15^, making it suitable for our study.

## Results

### Overall study design, data generation, and cell type identification

**Fig. 1:**
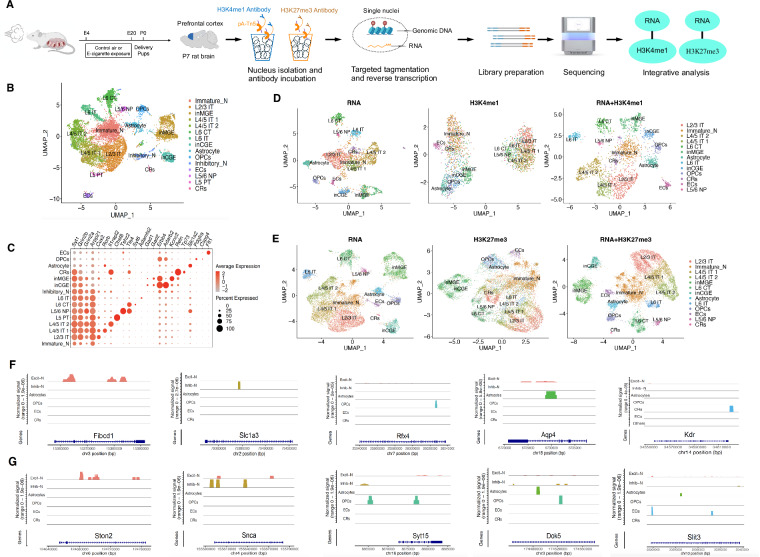
Study design, dataset characterization and cell type identification. **A** Overview of study design. Details can be found in the Results and Methods sections. The diagrams of rat brain and Paired-Tag flow were created with BioRender.com. **B** UMAP visualizing 15 cell types clustered from 14,170 single nuclei of P7 rat brain PFC. OPCs oligodendrocyte progenitor cells, ECs endothelial cells, CRs Cajal-Retzius cells, IT intratelencephalic excitatory neurons, PT pyramidal tract excitatory neurons, NP near projecting excitatory neurons, CT corticothalamic excitatory neurons. **C** Dot plot showing the expression of representative marker genes across all 15 cell clusters derived from P7 rat brain PFC. UMAP showing the integrated analysis of the single-nucleus transcriptomic and histone modalities of H3K4me1 (**D**) and H3K27me3 (**E**) Paired-Tag datasets, respectively. From left to right, transcriptome profiles, histone modification profiles, and integrated/combined snRNA-seq and histone modalities. Histone markers of the major cell types in H3K4me1 modification (**F**) and H3K27me3 modification (**G**).

In this study, we simultaneously profiled histone modifications (H3K4me1 and H3K27me3) and transcriptome from the same single nuclei using the Paired-Tag technology. The workflow for this study is shown in Fig. 1A. Briefly, pregnant rats were exposed to either e-cig vapor or control air from gestation day 4 (E4) to gestation day 20 (E20), and prefrontal cortices were harvested from postnatal day 7 (P7) offspring brains. This study included four groups: female control (F_Ctr), female E-cig (F_Ecig), male control (M_Ctr), and male E-cig (M_Ecig). The nuclei were isolated from a pool of prefrontal cortices obtained from four pups derived from two dams in each group. The Paired-Tag experiments were conducted according to the procedure as described previously^13^ with some modifications as detailed in the method section. Histone methylation data were processed at 5k bin and integrated with transcriptomic data from the same cell using R package Signac^16^.

After integration of histone modification and gene expression data and quality filtering, we recovered 2,544 and 11,626 nuclei with matched DNA and RNA profiles in H3K4me1 and H3K27me3 dataset, respectively (Supplementary Fig. 1A). Up to ~15,000 unique loci (5 kb bin) were mapped per nucleus for histone H3 modification (median number of unique loci per nucleus, 3124 for H3K4me1 and 4669 for H3K27me3, respectively) and up to ~3000 unique genes (nFeature_RNA) per nucleus were detected for RNA profiles (median number of genes: 954 for H3K4me1 and 866 for H3K27me3 dataset, respectively) (Supplementary Fig. 1A). The capture efficiency for RNA profile is on par with our previous snRNA-seq assay^5^. In both Paired-Tag datasets, we obtained comparable RNA and DNA profiles for each group (F_Ctr, F_Ecig, M_Ctr, and M_Ecig) (Supplementary Fig. 1A).

First, we performed independent cell clustering based on the transcriptomic profiles of 11,656 nuclei combined from the two Paired-Tag datasets (Fig. 1B, and Supplementary Data 1). Fifteen clusters were identified and annotated using cell type-specific markers (Fig. 1C). Most of the cells were identified as neurons (90.76%). Specifically, seven clusters showed high expression of excitatory neuron marker *Arpp21*^5,17^ in more than 50% of their cell population, whereas 3 out of 15 clusters showed high expressions of inhibitory neuron markers *Erbb4, Gad1*, or *Gad2*^5,17^ (Fig. 1C). We further identified seven cortical excitatory neuron subtypes based on established neuron subtype markers^5,18,19^. These subtypes include the common subgroups of projection neurons: the intratelencephalic (IT) excitatory neurons of different cortical layers, the pyramidal tract (PT) excitatory neurons, the near projecting (NP) excitatory neurons, and the corticothalamic (CT) excitatory neurons. The remaining four types of non-neuronal cells (9.24%) were identified as astrocytes (marker: *Slc1a2*), oligodendrocyte progenitor cells (OPCs) (markers: *Pdgfra* and *Cspg4*), Cajal-Retzius cells (CRs) (markers: *Reln* and *Tp73*), endothelial cells (ECs) (marker: *Flt1*), respectively. In both datasets, we found that the number of genes and histone peaks detected in each cluster largely depended on the cell identity and neurons  exhibited more complex gene expression and histone methylation (Supplementary Fig. 1B).

Next, we clustered the nuclei based on single-cell histone marks (H3K4me1 and H3K27me3, respectively). For consistency, we first conducted an unbiased cell clustering analysis using the snRNA-seq data alone as reference and then clustered the nuclei into the same number of clusters as obtained from the combined snRNA-seq data (11,656 nuclei), as shown in Fig. 1B. H3K4me1 mark clustered the 2,544 nuclei into two major clusters, one mainly contained excitatory neurons, and the others were mainly inhibitory neurons and non-neurons (Fig. 1D). H3K27me3 mark clustered the 11,626 nuclei at a much better resolution compared to H3K4me1, as good as observed in its matched snRNA-seq data (Fig. 1E). The underperformance of H3K4me1 on clustering single nuclei was possibly due to the fact that H3K4me1 has a widespread presence at enhancers, in contrast to the more localized and enriched presence of H3K27me3 at genes specific to the developmental processes or cell identity^20^. However, it should be noted that more H3K27me3 features were captured in each cluster as opposed to H3K4me1, which might improve the nucleus clustering (Supplementary Fig. 1B, C). Next, we integrated the transcriptomic data with the matched histone mark modality in the two Paired-Tag datasets and clustered the nuclei with Weighted Nearest Neighbors (WNN) method. As expected, in the H3K4me1 dataset, the nuclei were clustered similarly as using the transcriptomic data alone (Fig. 1D); whereas in H3K27me3 dataset, adding histone mark modality improved the clustering resolution, specifically enhanced the separation of excitatory neurons (Fig. 1E). Next, we screened for distinct histone modifications on genes with key biological functions to identify cell-type specific H3K4me1 and H3K27me3 markers. Considering that H3K4me1 in our data was not able to fully resolve the neuron subtypes, we combined all excitatory neurons into one group and all inhibitory neurons into another group. In sum, we identified cell type-specific H3K4me1 modifications for six major cell types and many of these distinct H3K4me1 marks are on the genes known as cell markers based their expression, e.g., *Fibcd1* for excitatory neuron^13^, *Slc1a3* for inhibitory neuron, *Rfx4* for OPCs^21^, *Apq4* for astrocytes^13,21^ and *Kdr* for CRs (Fig. 1F). For H3K27me3 modification, we proposed *Ston2* as an exclusive excitatory neuron marker (Fig. 1G**)**, which plays a key role in synaptic vesicle recycling at the synapse. We defined *Snca*, which encodes *alpha*-synuclein, as the inhibitory neuron H3K27me3 marker, which is known to regulate synaptic vesicle trafficking and neurotransmitter release at synapse. Similarly, we identified *Syt15* as a maker for OPCs; *Dok5* as a marker for astrocytes and OPCs; and *Slit3* as a marker for ECs (Fig. 1G).

### Prenatal e-cig aerosol exposure altered H3K4me1/H3K27me3 and neuron differentiation

**Fig. 2:**
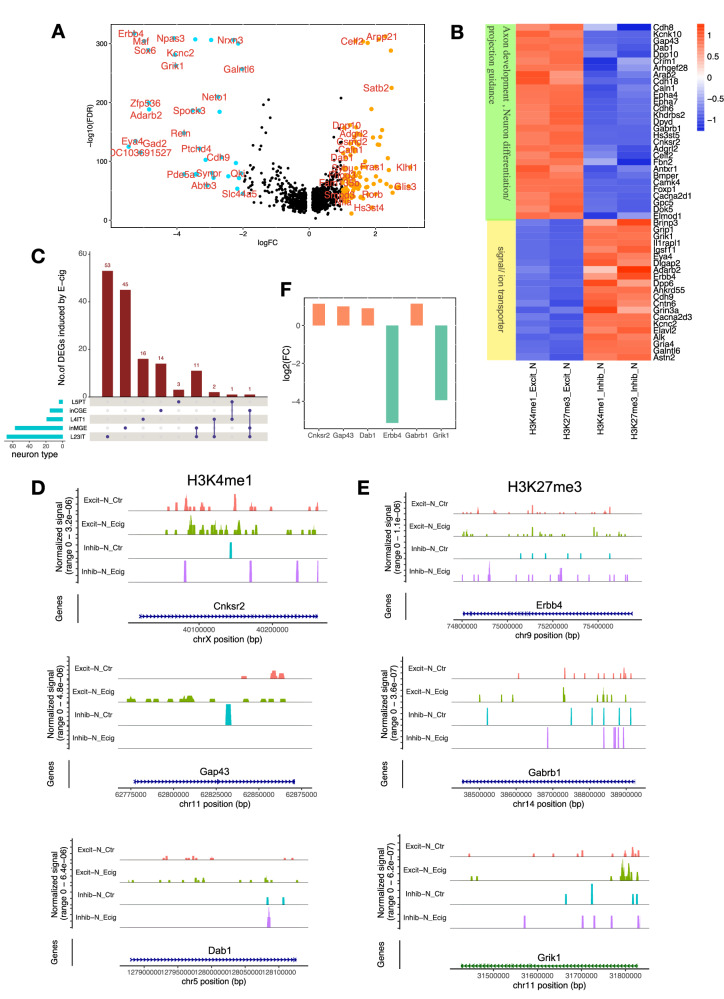
Regulation of neuron differentiation by H3K4me1 and H3K27me3 and the effects of prenatal e-cig exposure. **A** Volcano plot showing the differentially expressed genes (DEGs) between excitatory and inhibitory neurons in control PFC. Blue and red dots indicate the significant DEGs (|log2FoldChange| ≥1). **B** Heatmap showing the gene expression of the top 56 common DEGs between excitatory and inhibitory neurons identified from the H3K4me1 and H3K27me3 captures. **C** UpSet plot showing the number of DEGs identified in (**A**) that overlapped with the cluster DEGs induced by prenatal e-cigarette exposure. Peak coverage plots showing the signal distribution for H3K4me1 mark on *Cnksr2*, *Gap43*, and *Dab1* (**D**) or H3K27me3 mark on *Erbb4*, *Gabrb1*, and *Grik1*(**E**) in excitatory and inhibitory neurons, respectively. **F** Relative gene expression levels (excitatory neuron vs. inhibitory neuron) of the six genes shown in (**D**, **E**). All data used in (**A**, **B**) were from the control PFC samples only to exclude the potential effect of prenatal e-cig exposure.

It also has been documented that specific histone methylations are associated with cell-type-specific transcriptional activation or repression^20,22,23^. However, it was unknown whether histone methylations are regulated by prenatal e-cig aerosol exposure during rat brain development. In this study, we intended to identify the specific methylation patterns on H3K4 and H3K27 in excitatory and inhibitory neurons, and to determine if these neuron-type-specific histone methylation patterns could be altered by prenatal e-cig aerosol exposure in P7 rat prefrontal cortex. To increase the statistical power, we grouped 7 clusters of the excitatory neurons from the control group as total excitatory neurons and 3 clusters of the inhibitory neurons from the control group as total inhibitory neurons, based on the annotation displayed in Fig. 1B. We found that the excitatory neurons identified by H3K4me1 or H3K27me3 showed similar expression patterns on selected neuron marker (Supplementary Fig. 2A) and the differentially expressed genes (DEGs) between excitatory and inhibitory neurons from H3K4me1 and H3K37me3 datasets showed consistent enrichment in the biological processes, such as neuron differentiation and neuron development (Supplementary Fig. 2B), indicating the uniformity of the RNA-seq data from these two captures; thus, we merged the excitatory or inhibitory neurons from the two datasets. Using the merged cells, we identified 1,306 DEGs (p_adj< 0.01) between excitatory and inhibitory neurons, and 426 of them had |log2FC|> 0.5 (Fig. 2A and Supplementary Data 2). Gene ontology (GO) enrichment revealed that, in the excitatory neurons, the highly expressed genes were mainly involved in neurogenesis and axonogenesis, while the suppressed ones were ion and/or neurotransmitter transporters (Fig. 2B and Supplementary Fig. 3A). We overlapped those genes identified in Fig. 2A with the DEGs induced by prenatal e-cig aerosol exposure in several major types of neurons and found that 205 out of the 1306 DEGs between excitatory and inhibitory were affected by prenatal e-cig aerosol exposure in a cell type specific manner (Fig. 2C). For example, in Layer 2/3 intratelencephalic (IT) excitatory neurons, 64 genes were modulated by both cell differentiation signal and e-cig aerosol exposure, and 53 of these 64 genes were unique DEGs to Layer 2/3 IT. The expression changes of many excitatory or inhibitory neuron-specific genes were accompanied by changes in H3K4me1 and H3K27me3 profiles at the promoter regions, hinting prenatal e-cig aerosol exposure modulated gene expression through altering H3K4 and H2K27 methylations (Fig. 2D, E, Supplementary Fig. 3B, C, Supplementary Data 3).

We further examined the H3K4m1/H3K27me3 profiles and the biological functions of some neuron specific genes altered by prenatal e-cig aerosol exposure. Specifically, the H3K4me1 patterns of a few representative genes were illustrated in Fig. 2D. *Cnksr2* encodes a protein that plays a key role in the assembly of synaptic proteins at the postsynaptic membrane and is involved in dendritic development^24^. *Gap43* is highly expressed during axonal regeneration and is involved in synaptic transmission and plasticity, such as long-term potentiation and memory formation^25^. *Dab1* is a key component of the Reelin signaling and is essential for neuronal migration, dendrite outgrowth, and the positioning of neurons in the brain^26^. All the three genes illustrated in Fig. 2D showed higher H3K4me1 signal in excitatory neurons relative to inhibitory neurons, and enhanced signal intensity induced by prenatal e-cig aerosol exposure in excitatory neurons (Fig. 2D). The higher H3K4me1 signal in excitatory neurons was positively correlated with increased expressions (Fig. 2B). We also found that prenatal e-cig aerosol exposure altered the H3K27me3 profiles of many genes critical to neuronal functions (Fig. 2E). For examples, *Erbb4* is involved in the development of the central nervous system, including the cerebellum, and in the development and maturation of synapses^27^. *Gabrb1* encodes the *beta* 1 subunit of *gamma*-aminobutyric acid (GABA) A receptor, a chloride channel involved in the cellular response to histamine and the development of neurons^28^. *Grik1* encodes Glutamate Ionotropic Receptor Kainate Type Subunit 1, which is a ligand-gated ion channel that play a key role in excitatory neurotransmission and synaptic function^29^. Although the difference in H3K27me3 between the excitatory and inhibitory neurons was less dramatic, noticeably there were more H3K27 triple methylation in *Erbb4* and *Grik 1* genes in excitatory neurons, especially in the promoter regions (Fig. 2E**)**, which correlated with the suppressed expressions (Fig. 2B) in *Gabrb1* gene, we observed a weaker H3K27me3 signal (Fig. 2E) and a higher expression in the excitatory neurons (Fig. 2B). All in all, our results suggested that prenatal e-cig aerosol exposure regulated the expression of many genes involved in neuronal differentiation by altering the H3K4me1 and H3K27me3 profiles at gene promoters.

### snRNA-seq revealed prenatal e-cig aerosol exposure affected gene network and pathways involved in neuronal functions

**Fig. 3:**
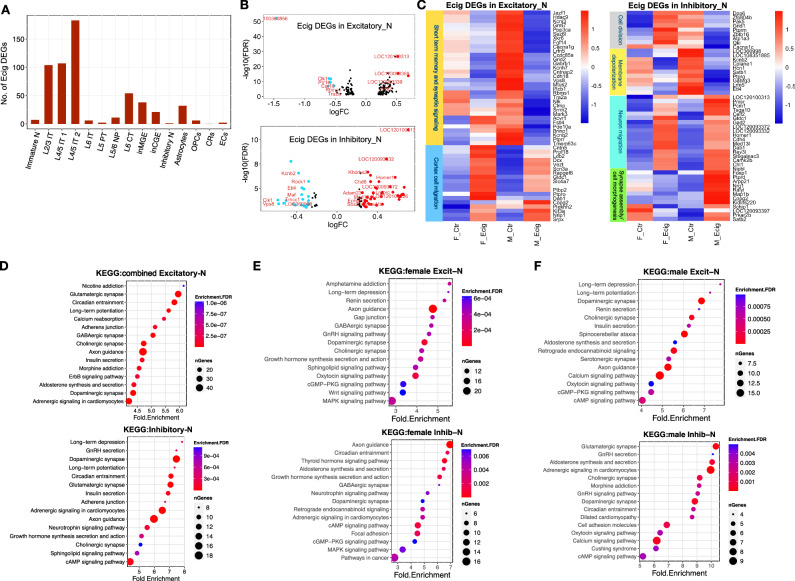
Prenatal e-cigarette exposure altered gene network and pathways involved in neuronal function. **A** Bar plot showing the number of DEGs induced by prenatal e-cigarette exposure in each cell type. **B** Volcano plots showing the DEGs in excitatory neurons or inhibitory neurons. Blue and red lines indicate |log2FoldChange| ≥0.3. **C** Heatmaps showing the DEGs in excitatory or inhibitory neurons. Dot plots showing the Kyoto Encyclopedia of Genes and Genomes (KEGG) pathways enriched based on the DEGs in excitatory or inhibitory neurons from female and male combined (**D**), female alone (**E**), and male alone (**F**).

Differential gene expression analysis on each cell type (identified in Fig. 1B) showed that the cortex L2/3 IT and L4/5 IT excitatory neurons had the most DEGs (FDR < 0.1, |log2FC|> 0.25) (Fig. 3A and Supplementary Data 4). To reach a better statistical conclusion and minimize the number of false DEGs, we combined all excitatory neurons into one group and all inhibitory neurons into another group and performed DEG analysis. We identified 109 and 158 DEGs in excitatory and inhibitory neurons, respectively (Supplementary Data 4). We further used *p* < 0.001 and |log2FC|> 0.25 thresholds to select DEGs for biological functional enrichment. It is intriguing that the CDC-like kinase 1 (*Clk1)* gene was significantly suppressed in both excitatory and inhibitory neurons (Fig. 3B). CLK1 protein is part of molecular clock that regulates circadian rhythms^30^. Nicotine, the primary e-cig component, has been shown to cause sleep disturbances and lead to irregular circadian rhythms, snoring, and obstructive sleep apnea^31^. Other DEGs involved in circadian regulation include *Cacna1c*, *Camk2b*, *Prkcb*, *Gria4*, *Adcy1*, *Rps6ka5*, and *Ryr2*. We speculate that prenatal e-cig aerosol exposure may affect offspring circadian rhythms and consequently the entrainment of biological processes. KCNB2 protein is active in GABAergic synapse and presynaptic membranes and regulates neuron excitability by controlling potassium ion outflow^32,33^. Our result showed that *Kcnb2* was significantly downregulated in inhibitory neurons by prenatal e-cig aerosol exposure (Fig. 3B), and the reduced KCNB2 expression may lead to a more positive membrane potential (depolarization) in GABAergic neurons. *Ptbp2*, which encodes the polypyrimidine tract binding protein, was significantly upregulated in excitatory neurons (Fig. 3B). PTBP2 governs the axonogenesis-associated alternative splicing necessary for robust generation of a single axon in mammals^34^. The expression of *Khdrbs2*, another gene involved in regulating alternative mRNA splicing, was markedly increased in inhibitory neurons (Fig. 3B). KHDRBS2 regulates the evolutionary conserved neurexin alternative spliced segment 4 (AS4), which is involved in neurexin selective targeting to postsynaptic partner and maintaining synaptic connections between neurons in brain^35^.

In both excitatory and inhibitory neurons, the upregulated genes were primarily involved in cell migration. In contrast, the downregulated genes in excitatory neurons were mainly associated with synaptic signaling and short memory, while the downregulated genes in inhibitory neurons were mainly involved in membrane potential (Fig. 3C). We also found that more genes exhibited sex difference in expression in response to prenatal e-cig aerosol exposure in inhibitory neurons compared to excitatory neurons (Fig. 3C). In addition, some key genes of signal receptor and transduction, such as *Acvr1*, *Fstl4*, *Pde10a*, and *Kcnq2*, were upregulated in excitatory neurons in female but were downregulated in male. In inhibitory neurons, the expression of many genes involved in cell division were increased in female but reduced in male; while the cell morphogenesis genes were induced in male but suppressed in female (Fig. 3C). In sum, our results suggested that prenatal e-cig exposure altered the neuron development, and it bring its impact to bear on male and female offspring differently.

In excitatory neurons, KEGG (Kyoto Encyclopedia of Genes and Genomes) pathways involved in axon guidance and synapse transduction were significantly enriched from e-cig aerosol induced DEGs (Fig. 3D). The long-term suppression of synaptic transmission and drug addiction related pathways was consistently enriched in both male and female offspring (Fig. 3E, F). In inhibitory neurons, the circadian entrainment and thyroid hormone pathways were significantly enriched (Fig. 3D–F). All in all, our data showed that prenatal e-cig aerosol exposure broadly impacts neuron synapse function, axon guidance, neuron migration, neuron morphogenesis, as well as many signaling pathways like cAMP and calcium signaling, indicating a profound detrimental effect on brain development. In addition, our data suggested that prenatal e-cig aerosol exposure disrupted the circadian entrainment in the offspring (Fig. 3D–F), which in turn may cause behavioral, metabolic, and physiological disturbances^36^.

### Prenatal e-cig aerosol exposure altered H3K4me1-H3K27me3 transition and gene bivalency involved in neuronal cell type specification

**Fig. 4:**
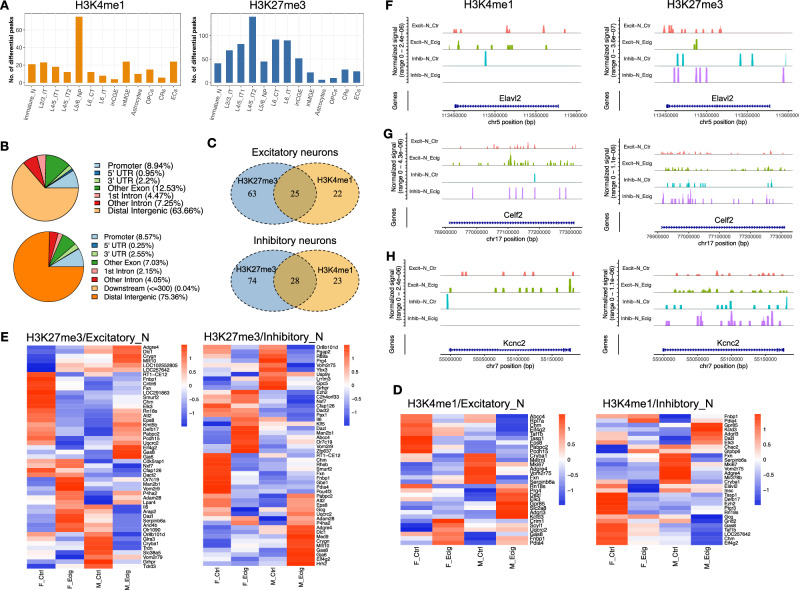
Prenatal e-cigarette exposure changed H3K4me1-H3K27me3 transition and bivalency in the genes involved in neuronal cell type specification. **A** Bar plot showing the number of differential histone peaks (5k bin) of H3K4me1 and H3K27me3 induced by prenatal e-cigarette exposure in each cell type. **B** Pie charts showing the distribution and localization of differential histone peaks of H3K4me1 (top) and H3K27me3 (bottom) in relation to genic regions. **C** Venn diagrams showing the number of genes with significantly differential methylation of H3K4me1 or/and H3K27me3 in excitatory neurons (top) or inhibitory neurons (bottom), respectively. Heatmaps showing the differential histone peaks (5k bin) of H3K4me1 (**D**) and H3K27me3 (**E**) in excitatory and inhibitory neurons, respectively. Histone mark signal was normalized to fragments per kilobase per million mapped reads (FPKM). Peak coverage plots showing the e-cig induced changes in H3K4me1 (**F**) and H3K27me3 (**G**) of three representative bivalent genes, *Elavl*2 (**F**), *Celf2* (**G**), and *Kcnc2* (**H**), in excitatory neurons and inhibitory neurons.

We have shown that prenatal e-cig aerosol exposure changes transcriptomic profiles and chromatin accessibility in many types of brain cells^5^. In this study, we found that prenatal e-cig aerosol exposure modulated H3K4me1 and H3K27me3 profiles in virtually all types of cells captured in our dataset, although it had a more pronounced effect on H3K27me3 than on H3K4me1 (Fig. 4A and Supplementary Data 5 and 6, Supplementary Fig. 4). Most of the differential peaks (DePeaks) (63.66% in H3K4me1 and 75.36% in H4K27me3, respectively) were annotated to the distal intergenic regions and about 8.5% of those were annotated to promoter regions (TSS ± 1 kb) in both H3K4me1 and H3K27me3 dataset (Fig. 4B). Given that the promoter regions only account for less than 2% of the total rat genome, our results revealed that prenatal e-cig aerosol exposure preferentially modulated H3 methylation at promoters, suggesting H3 methylation regulates gene expression. The remaining DePeaks were annotated to various genic categories with no significant hot spots in any regions other than promoter. From aggregated excitatory and inhibitory neurons, we identified 47 and 51 genes with DePeaks on H3K4me1 in excitatory and inhibitory neurons, respectively; and 88 and 102 genes with DePeaks on H3K27me3 in excitatory and inhibitory neurons, respectively (Fig. 4C and Supplementary Data 5 and 6). Many of those genes with differential H3 methylation harbored both H3K4me1 (enhancer) and H3K27me3 (suppressor) DePeaks (Fig. 4C and Supplementary Data 5 and 6), indicating bivalent regulation on those genes. Heatmaps of those DePeaks revealed similar H3 methylation changes with some subtle difference in H3K4me1 and H3K27me3 modifications in both excitatory and inhibitory neurons (Fig. 4D, E). We also observed that prenatal e-cig aerosol exposure induced different changes in H3K4m1 and H3K27me3 methylation between female and male. For instance, in excitatory neurons, *Eif4g2* and *Pgap2* showed opposite changes in H3K4me1 profile between male and female (Fig. 4D), while *Gpr89b*, *Pcdh15*, and *Or9s18* had opposite changes between male and female in H3K27me3 modification (Fig. 4E). We found that the bivalent genes with H3K4me1 and H3K27me3 DePeaks likely encode proteins of upstream signaling molecules like ADGRE4 or GAS8; or proteins controlling a metabolism pathway, like CES1F and FRATAXIN. A significant number of the bivalent genes with DePeaks have essential roles in central nervous system development, like *Fbnp1, Eif4g2*, *Chm*, and *Lamp1*. Furthermore, prenatal e-cig aerosol exposure also affected H3 methylation of genes involved in transcription and translation such as *Or7c19, Rn18*, and *Dazl* (Table 1 and Supplementary Data 5 and 6).

**Table 1:** Functions of bivalent genes with differential methylation pattern

Gene	Description	Function
*Adgre4*	Adhesion G protein-coupled receptor E4	Involved in adenylate cyclase-activating G protein-coupled receptor signaling pathway
*Ces1f*	Carboxylesterase 1F	Acts upstream of or within short-chain fatty acid catabolic process
*Cesl1*	Carboxylesterase-like 1	Enable carboxylesterase activity
*Chm*	CHM Rab escort protein	Involved in protein targeting to membrane and vesicle-mediated transport
*Dazl*	Deleted in azoospermia-like	Involved in positive regulation of translational initiation and meiotic nuclear division
*Eif4g2*	Eukaryotic translation initiation factor 4, gamma 2	Involved in positive regulation of axon extension, dendritic spine development, and translation
*Fnbp1*	Formin binding protein 1	Involved in nervous system development
*Fxn*	Frataxin	Involved in cellular response to glucose starvation and positive regulation of axon extension
*Gas8*	Growth arrest specific 8	Acts upstream of or within several processes, including brain development
*Lamp1*	Lysosomal-associated membrane protein 1	Involved in autophagic cell death, cytoplasmic vesicle trafficking. Active in synaptic vesicle membrane
*Or7c19*	Olfactory receptor family 7 subfamily C member 19	Acts upstream of or within regulation of DNA-templated transcription
*Pttg1ip*	PTTG1 interacting protein	Acts upstream of or within negative regulation of DNA damage and intrinsic p53 apoptotic signaling pathway
*Rn18s*	18S ribosomal RNA	Protein translation
*Serpinb6a*	Serpin family B member 6A	Involved in cellular response to osmotic stress and sensory perception of sound

Rat *Elavl2* encodes a member of the Elav-like RNA-binding proteins^37^, which regulates mRNA stability and translation by binding to specific RNA sequences. Elavl2 plays a key role in neuronal development, synaptic plasticity, and neuronal survival^38^. In this study, *Elavl2* showed strong bivalency and low expression in excitatory neurons (Fig. 4F, Supplementary Data 2), suggesting *Elavl2* promoter was in a poised state in control group. In inhibitory neurons, H3K4me1 signal in *Elavl2* was much lower in control group and was completely absent in e-cig group. Prenatal e-cig aerosol exposure increased H3K27me3 signal at *Elavl2* promoter in inhibitory neurons (Fig. 4F), which correlated with a reduced *Elavl2* expression in e-cig group (Supplementary Data 2). *Celf2* was another gene with strong bivalency that was altered by prenatal e-cig aerosol exposure (Fig. 4G), which plays a key role in controlling neural progenitor differentiation, neuronal identity, and neuron maturation by regulating the splicing in response to brain development^39^. Prenatal e-cig aerosol exposure also modulated the bivalency of genes essential for neuron functions, such as *Kcnc2*, which encodes a voltage-gated monoatomic ion transporter involved in cellular response to nitric oxide, nervous system development, and protein complex oligomerization^40^. *Kcnc2* was a bivalent gene with a poised promoter in P7 rat excitatory neurons, as both H3K4me1 and H3K27me3 were present in *Kcnc2* gene body and its promoter region (Fig. 4H). In conclusion, prenatal e-cig aerosol exposure dynamically changed H3K4me1 and H3K27me3 profiles in many genes in excitatory and inhibitory neurons, through which it altered the specific gene expression during CNS development.

### Prenatal e-cig aerosol exposure induced systematic changes in H3K4me1-H3K27me3 bivalency in excitatory neurons

**Fig. 5:**
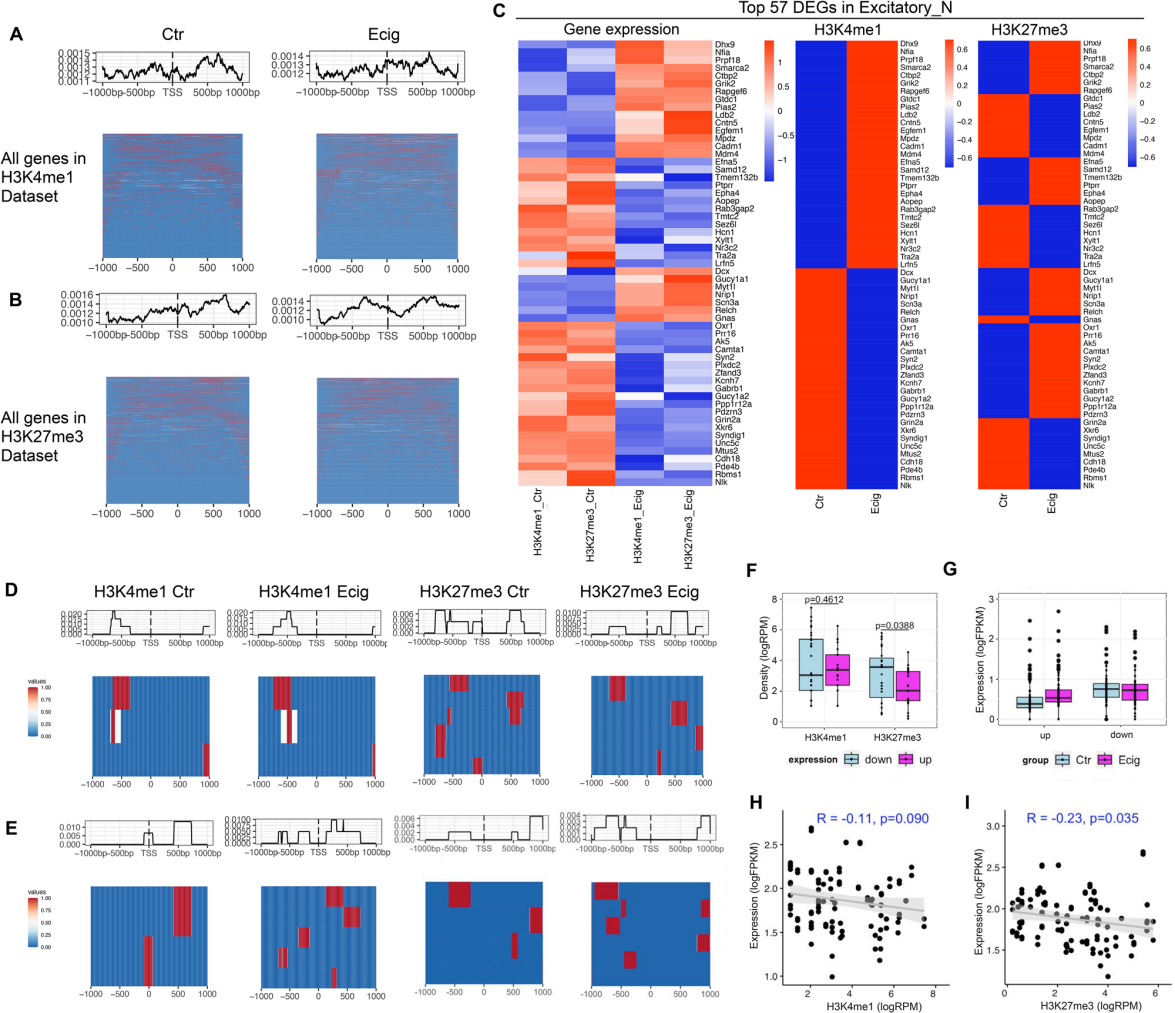
Prenatal e-cigarette exposure altered the gene expression in excitatory neurons through modifying H3K4me1-H3K27me3 bivalency at target genes. Intensity (top panel) and heatmap plots (lower panel) showing the H3K4me1 (**A**) and the H3K27me3 (**B**) profiles at promoter (TSS ± 1 kb) regions of all genes in all excitatory neurons. The top panel above heatmap shows the aggregated histone modification at the specific distance relative to TSS. **C** Heatmaps showing the expression of the top 57 bivalent DEGs identified in excitatory neurons, and the methylation signal intensity of H3K4me1 and H3K27me3 at the promoter (TSS ± 1 kb) regions of those DEGs. H3K4me1 and H3K27me3 signal intensity at promoter (TSS ± 1 kb) regions of the 73 up-regulated genes (**D**) and the 36 down-regulated DEGs (**E**) identified in excitatory neurons. The top panel above each heatmaps shows the aggregated histone modification at the specific distance relative to TSS. Boxplots showing the H3K4me1 and H3K27me3 signal intensity at the promoter regions (**F**) of those DEGs presented in (**C**), and their expression levels (**G**). Scatter plots with Pearson correlation test showing the correlation between the expression of DEGs and the H3K4me1 (**H**) or H3K27me3 (**I**) signal intensity at their promoter regions. *n* = 73 for upregulated genes and *n* = 36 for downregulated genes in (**D**–**G**). Histone mark signal was normalized to Reads Per Million mapped reads (RPM). Gene expression level was normalized to fragments per kilo base per million mapped reads (FPKM).

We further examined the systematic change of H3K4me1-H3K27me3 signature during early brain development, as well as under the influence of prenatal e-cig aerosol exposure, using excitatory neurons as an example. Our data showed a near bimodal distribution of H3K4me1 and H3K27me3 profiles around promoter regions (TSS ± 1 kb) of all genes detected (Fig. 5A), which represented an average modification signal. The signals were consistently stronger around 500 bp downstream of the TSS. Excitatory neurons in e-cig group exhibited similar H3K4me1 methylation profile compared to those of control (Fig. 5A). Slightly increased H3K27me3 signals were found 300 bp upstream of TSS in e-cig samples compared to control (Fig. 5B). Overall, there were no consistent correlations between the H3K4me1 or H3K27me3 signal intensity in the gene body and the expression levels of the top 57 DEGs induced by prenatal e-cig aerosol exposure (Fig. 5C). Specifically, about two thirds of the upregulated DEGs showed an increased H3K4me1 signal whereas the other one third of the upregulated DEGs showed a reduced H3K4me1 signal in their gene bodies. Slightly less than two thirds of the downregulated DEGs had a reduced H3K4me1 signals, whereas the remaining showed an elevated signal. On the other hand, we found that the majority of the DEGs (47.37%) exhibited an opposite change between the signal of H3K27me3 mark and the gene expressions (Fig. 5C).

We observed a unimodal distribution of H3K4m1 signal 500 bp upstream of TSS from data compiled from all up-regulated DEGs in both e-cig and control samples (Fig. 5D). There was no change in H3K4me1 intensity, rather than a slightly wider distribution in e-cig group compared to the control. In the down-regulated DEGs, the H3K4me1 signal shifted to downstream 500 bp region in the e-cig group, suggesting less occupancy by transcription factors, but more presence of H3K4 methylation complex (Fig. 5E). There was no significant difference in the H3K4me1 signal intensity between the up-regulated or down-regulated DEGs (*p* = 0.4612) (Fig. 5F). Pearson correlation analysis revealed a low but not significant negative correlation between H3K4me1 signal strength and the gene expression for the DEGs (*R* = −0.11, *p* = 0.090) (Fig. 5H). These results suggested that H3K4me1, at least on its own, may not play an important role in regulating the expression of DEGs. We observed a remarkably reduced H3K27me3 signal at the 500 bp to 1000 bp region upstream of TSS in the upregulated DEGs (Fig. 5D), but an increased signal in the promoter regions of the downregulated genes (Fig. 5E). In addition, the H3K27me3 signal strength was significantly reduced in the up-regulated DEGs than that in the down-regulated DEGs (*p* = 0.0388) (Fig. 5F). The Pearson correlation analysis revealed a low yet significant (*R* = −0.23, *P* = 0.035) negative correlation between the expression level of those DEGs and the H3K27me3 signal at their promoters (Fig. 5I). These results suggested that H3K27me3 plays a more important role than H3K4m1 in regulating the transcription of those DEGs induced by prenatal e-cig aerosol exposure. Although the loss of H3K27me3 signal was associated with the activation of transcription, it did not necessarily correlate with the copy numbers of the transcripts i.e., the downregulated genes showed stronger H3K27me3 presence at TSS regions, but higher transcription levels than the upregulated DEGs (Fig. 5F, E). In sum, our data supported the view that prenatal e-cig exposure modulates the transition from H3K27me3 to H3K4me1 which orchestrates the neuron specific gene expression in the PFC of the developing rat brain.

## Discussion

Histone methylation influences the accessibility of DNA to transcription factors and RNA polymerase by altering the structure of chromatin, thereby regulating gene expression^6,7,23^. This dynamic interplay between histone methylation and transcriptional regulation is essential for the central nerve system development, including neuron differentiation, axon guidance, synaptogenesis, etc^35^. Using snRNA-seq and snATAC-seq technology, we previously demonstrated that prenatal e-cig aerosol exposure altered neuron transcription and chromatin accessibility of P7 rat brain, consequently altered excitatory and inhibitory neuron differentiation and their balance^5^. In this study, we applied Paired-Tag technology to simultaneously profile the transcriptome as well as H3K3me1 and H3K27me3 modifications within the same single nuclei isolated from prefrontal cortex of P7 rat prenatally exposed to e-cig aerosol. We identified all the major types of cells in prefrontal cortex, such as excitatory neurons (71.37%), inhibitory neurons (16.94%), astrocytes (4.30%), oligodendrocytes (3.40%), ECs (3.04%), and CRs (0.94%) (Fig. 1B). Our results were in line with the fact that excitatory neurons are the major neuronal type in the cerebral cortex^41^ and 90% of all captured cells were either excitatory or inhibitory neurons, which is on a par with other similar studies using Paired-Tag technology that reported a higher and comparable recovery rate of neurons from similar brain regions^13^.

Unsupervised clustering using histone data alone showed a large discrepancy between H3K4me1 and H3K27me3 in their ability to distinguish cell types. The H3K4me1 profiling clustered the 2544 nuclei into two major clusters, one mainly consisted of excitatory neurons and the other mainly contained inhibitory neurons and non-neurons; the H3K27me3 profiling distinguished the most cell types identified in snRNA-seq data (Fig. 1E). The underperformance of H3K4me1 on classifying cell types was possibly due to the less specific distribution of H3K4me1 across a wide range of enhancers shared by many cell types^42^. Although integrating single nucleus histone modality with snRNA-seq data did not remarkably improve cell clustering resolution for H3K4me1 dataset, our data did show the feasibility to identify brain cell types using H3K27me3 profile alone in a complex brain cell population (Fig. 1F, G). At a reduced cell complexity, high specific bivalent histone marks could be linked to cell type specific gene expression (Fig. 2B, D, E), making it possible to understand neuron type specific transcriptional regulation from histone modifications. Studies have shown that the disruption of the circadian entrainment is frequently associated with drug addiction^43^ and is considered a risk factor for the development of addictive disorders^44^. The circadian regulation of reinforced behaviors is determined both genetically, *i.e*., mutations in major circadian regulators^30^ and epigenetically through the entrainment from the environment clues, such as light and drugs^36,43,44^. Nicotine and the major solvents in e-cigarette, such as propylene glycol have been shown to affect the expression of genes involved in the circadian rhythm^45^ and sleep^46^, on the other hand, sleep deprivation can increase nicotine use in humans^47^. In this study, we found that the circadian rhythm pathway was significantly enriched by prenatal e-cig exposure induced DEGs in both male and female inhibitory neurons (Fig. 3E, F), suggesting that prenatal e-cig aerosol exposure not only disturbed the neonatal rat circadian rhythms but also imprinted this adverse influence epigenetically, impacting the biological rhythm at later stage even after the cessation of exposure. Thus, our findings offered an epigenetic clue about the long-term adverse effect of prenatal e-cig aerosol exposure on offspring brain development. Genes enriched in circadian entrainment included calcium signaling genes *Cacna1c*, *Camk2b*, and *Ryr2*; cAMP and protein kinase signaling gene *Adcy1* and *Prkcb*; as well synaptic transmitter receptor *Gria4*, indicating that prenatal e-cig aerosol exposure altered P7 rat circadian entrainment at a wide spectrum, which was also supported by a recent study^31^.

Bivalent histone marks consist of large regions of H3 lysine 27 methylation and smaller regions of H3 lysine 4 methylation, which prepare the key developmental genes to be rapidly switched-on in a cell type or tissue specific manner during differentiation and development^20,48,49^. In this study, we found that prenatal e-cig aerosol exposure changed the neuron differentiation and maturation during the early rat brain development by modulating bivalent fingerprints of H3K4me1 and H3K27me3. At genome level, regardless of the expression, our data showed a near bimodal distribution of H3K4me1 and H3K27me3 signal at either side of all known TSS, with a slightly stronger signal around 500 bp downstream of the TSS (Fig. 5A), which suggested that most promoters in the neurons were in a silenced state. We did not observe significant association of the H3K4me1 pattern around TSS with the gene expression change (Fig. 5H), indicating that the presence of H3K4me1 at promoter was not a prerequisite for an active transcription. Conversely, H3K27me3 signal intensity around TSS exhibited small yet significant negative correlation with the gene expression level (Fig. 5I). This is likely because H3K27me3 is more pervasive in suppressing the transcription, and the removal of this histone mark is required to transit the gene into an active state. In line with our findings, it has been known that the bivalent H3K27me3 mark was lost or diminished in the promoters for many neural genes during inactive to active transition status^20,50^, while H3K4me1 signature remained no change^51^. Our data support the view that bivalent chromatin does not poise the genes for rapid activation but protects the promoters from a de novo DNA methylation^48,52^. In conclusion, our observations supported the view that, during neuronal differentiation, bivalent promoters undergo H3K27me3-H3K4me1 transition, and the loss of H3K27me3 is accompanied by a bimodal pattern loss or unimodal pattern enrichment of H3K4me1^49^. Prenatal e-cig aerosol exposure affected the bimodal transition and expression of a small number of cell type specific genes^53^ which determine the excitatory and inhibitory neuron differentiation during the early rat brain development.

## Methods

### Prenatal e-cigarette exposure animal model

All animal experiments involving animal care, surgery, and sample preparation were approved by the Institutional Animal Care and Use Committee of Loma Linda University. We have complied with all relevant ethical regulations for animal use. Four pregnant Sprague-Dawley (SD) rats, 3 months old, were purchased from Charles River Laboratories (Portage, MI) and were randomly divided into two groups: two for e-cigarette exposure group and two for control group; the e-cigarette exposure group was exposed to e-cigarette aerosol; the control group was exposed to air in the same type of chamber. Pregnant SD rats were housed under controlled temperature (22 °C) and photoperiod (12 h light and 12 h dark cycle) with food and water ad libitum both light and dark phases.

We used a chronic intermittent e-cigarette (CIEC) exposure model in pregnant rats as previously described^5^. The e-cigarette rodent exposure system was manufactured by AutoMate Scientific, Inc. (Berkeley, CA). This system contains air pressure and flow rate control as well as hardware and software that allow experimenters to control the timing, duration, and times/day for CIEC aerosol generation and exposure in a free-moving rodent exposure chamber. The commercial (BluPlus Cig) e-cigarettes containing 2.4% nicotine were used for this project to reflect what real-world e-cigarette users are experiencing. All e-cigs and supplies were purchased on the bluCig website. Batteries were charged every day and were replaced every other week during the experiments. During the light phase of 12 h time (09:00–21:00), rats were returned to their home cages without aerosol delivered. To mimic the phenomenon of chronic intermittent e-cigarette exposure in human vaporizers, we activated 1 e-cigarette every time, adjusted the air pressure to 9.5 psi, and adjusted the air-flow rate to 4.5 L/min. Our CIEC exposure protocol was: puff duration of 4 s, 3 puffs in an inter-puff interval of 30 s per vaping episode, and one episode per 1 h in the dark phase of 12 h each day, which generates similar nicotine blood pharmacokinetics in the pregnant rats to those observed in human e-cigarette users^54,55^. The dams were exposed to e-cigarettes for a total of 17 days from gestational or embryonic day 4 (E4) to E20.

### Brain tissue dissection

Two male and two female postnatal day 7 (P7) rats from dams exposed to either e-cigarette aerosol or control air were selected for this experiment (4 pups per dam, totally 16 pups from 4 dams). Briefly, P7 rat pups were euthanized by decapitation under deep isoflurane anesthesia. The prefrontal cortex was isolated on ice from the whole brain as previously described^56^. We harvested 16 P7 brain prefrontal tissues from four groups consisting of male control, male e-cig, female control, and female e-cig, i.e., four P7 brain prefrontal cortexes (2 from each dam) were pooled together in each group for Paired-Tag experiment.

### Paired-Tag procedure

The Paired-Tag experiment was carried out with the assistance provided by Epigenome Technologies, Inc. (San Diego, CA), following the protocol as described previously^57^. In brief, nuclei were isolated using 10–30 mg of prefrontal cortex tissues obtained from the P7 SD rat brains, permeabilized with antibodies that recognize specific histone modifications (2 µg antibody in 50 µl volume) and guide the binding of protein A-fused Tn5 transposase to chromatin. Antibodies against H3K4me1 and H3K27me3 were purchased from Abcam (Cambridge, UK) and EpiCyphter (Durham, NC), respectively. Then, sequential tagmentation and reverse transcription (RT) were carried out. The reactions took place across 12 wells, each containing a unique DNA barcode integrated into the transposase adaptors and RT primers, enabling sample- or replicate-specific labeling (first round of barcoding). Subsequently, a ligation-based combinatorial barcoding method was employed to introduce the second and third rounds of barcodes. This step involved attaching well-specific DNA barcodes to the 5’-ends of chromatin DNA fragments and cDNA generated from the RT in 96-well plates. Finally, the barcoded nuclei were divided into sub-libraries, lysed, and the chromatin DNA and cDNA were purified, amplified, split into two separate sequencing libraries, one dedicated to each modality. The libraries were sequenced on an Illumina NextSeq 2000 at 150 × 2 bp, paired-ended read. We obtained the following reads for each of the two modalities (DNA or RNA) presented as raw reads per single nucleus, H3K4me1: DNA, 29.659 K, RNA, 32.269k; H3K27me3: DNA, 36.466k, RNA, 26.462k (Supplementary Fig. 1).

### Preprocessing of Paired-Tag data

Paired-Tag data was preprocessed following the methods as described previously^13^. In brief, cellular barcodes and the linker sequences were read by Read 2. The first bases of barcode (BC) no. 1, BC no. 2, and BC no. 3 should be located within the 84th–87th, 47th–50th and 10th–13th bases of Read 2. We identified the positions of barcodes by matching the linker sequences adjacent to the cellular barcodes. A bowtie reference index was generated with all possible cellular barcode combinations (96 × 96 × 12), and barcode sequences were mapped to the cellular barcode reference using bowtie^58^ with the parameters: *-v 1 -m 1 --norc* (reads with more than 1 barcode mismatch which can be assigned to more than 1 cell were discarded). NextEra adaptor sequences were trimmed from 3′ of DNA and RNA libraries, Poly-dT sequences were further trimmed from 3′ of RNA libraries and low-quality reads (minimal length: *L*  =  30, minimal base calling quality: *Q*  =  30) were excluded from further analysis.

### Reads mapping and quality control

Cleaned reads were first mapped to *Rnor* 7.0 reference genome with STAR^59^ (v.2.6.0a) for RNA or bowtie2^60^ for DNA. Mapped DNA reads of H3K4me1and H3K27me3 were further filtered by MAPQ  >  10. Duplicates were removed based on the mapped position, cellular barcode, PCR index, and UMI. We used BC no. 1 for the identification of the origin of samples. Low-coverage nuclei were removed from further analysis (<200 transcripts and <500 unique DNA reads). Before generating the cell-counts matrices, DNA bam files were further filtered by removing high-pileup positions (cutoff = 10) regardless of cellular barcode, PCR index and UMI.

### Processing of Paired-Tag data

RNA alignment files were converted to a matrix with cells as columns and genes as rows. DNA alignment files were converted to a matrix with cells as columns and 5 kb bins (instead of peaks) as rows. Cells with <200 features in RNA and <500 features in DNA bin were removed. The DNA matrix was further filtered by removing the 2% highest covered bins. The clustering of single nuclei based on RNA profiles was performed using the Seurat package 5.0^61^. Briefly, cell-to-gene counts were normalized, and variable genes were selected for dimension reduction by Principal Component Analysis (PCA), visualized with UMAP and clustered with the Louvain algorithm. Cell groups with high expression levels of marker genes from multiple major cell types were considered as doublets and excluded from further analyses. Single-cell DNA profile was analyzed using Signac^16^. Specifically, cell-to-bin (5-kb bin size) matrices were binarized and normalized by Run Term Frequency Inverse Document Frequency (TF-IDF) method, then followed by dimension reduction by PCA, visualization with UMAP on “lsi” reduction. To cluster single nuclei with joint modalities, snRNA-seq data and DNA cell-to-bin data were first subject to dimension using “pca” or “lsi”, respectively. Then, FindMultiModalNeighbors function was used to integrate the two modalities using the first 30 components for “pca” and second to 30th components for “lsi”. Finally, cells were visualized with UMAP using Weighted Nearest Neighbor (WNN) analysis.

### Differential analysis of single cell gene expression and histone modification

After dimension reduction, molecularly distinct clusters were identified using FindClusters function with the original Louvain algorithm. This allowed us to identify 15 cell types in the combined snRNA-seq dataset. Cluster marker genes were identified using FindMarkers function, and those marker genes were cross-referenced with known cell markers to identify cell types. Differentially expressed genes (DEGs) and differential peaks between prenatal e-cig exposure and control groups were determined using FindMarkers function and significance was defined by “MAST” method with FDR < 0.01 and |fold change|>1.25. All Seurat-generated clusters containing less than 50 cells were excluded. Differential peaks were annotated to *Rnor* 7.0 genome using annotatePeak function from R package CHIPseeker^62^.

### Canonical pathway and molecular function analysis

Analyses of the gene bio-functional pathways were performed using online analysis tool ShinyGo (v8.0, http://bioinformatics.sdstate.edu/go/). We also applied ShinyGO enrichment tool to the lists of DEGs and differential peak linked genes to identify the molecular functions and biological processes that may be regulated by maternal e-cigarette exposure at transcriptomic level.

### Statistics and reproducibility

All data are presented as mean ± SD. Cell proportion was calculated by combining the two subgroups (female and male) in each treatment group (e-cigarette and control), respectively. To compare the difference, *p* values were calculated using two-tailed Student’s *t* test with a significant level of 95%. For single nucleus RNA-seq and histone data, four animals (from two dams) were pooled in each group (female control, male control, female e-cigarette, and male e-cigarette). The differential expression or differential 5 kb bin peak was determined using a non-parametric Wilcoxon rank-sum test as part of the Seurat package. Both the p-value and the adjusted p-value were reported in the single nucleus sequencing data.

### Study limitations

While this study provides valuable insights into understanding the early rat brain development under the influence of prenatal e-cig aerosol exposure, the sample size was relatively small, four P7 animals from two dams in each group. This may limit the generalizability of the findings. Future research would aim to include a larger and more diverse population to enhance the robustness of the results.

### Reporting summary

Further information on research design is available in the Nature Portfolio Reporting Summary linked to this article.

## Supplementary information

Supplementary Information

Description of Additional Supplementary Files

Supplementary Data 1

Supplementary Data 2

Supplementary Data 3

Supplementary Data 4

Supplementary Data 5

Supplementary Data 6

Supplementary Data 7

Reporting Summary

## Data Availability

All data generated, i.e., the Paired-Tag single-cell histone mark methylations (H3K4me1 and H3K27me3) and snRNA-seq are available in the GEO, accession # GSE280559, with the following link: https://www.ncbi.nlm.nih.gov/geo/query/acc.cgi?acc=GSE280559. Other relevant data are available from the corresponding author as requested.

## Code availability

All Paired-Tag data preprocessing codes used are available on Github from https://github.com/cxzhu/Paired-Tag. Differential histone peak and gene expression were analyzed using Seurat (https://github.com/satijalab/seurat) and Siganc codes (https://github.com/stuart-lab/signac).
