# Supplementary material for: Single-cell profiling of H3K4me1-H3K27me3 revealed bivalent regulation of abnormal neuronal development caused by prenatal e-cigarette vaporing
Source: Commun Biol. 2025 Sep 1;8:1326. doi: 10.1038/s42003-025-08683-8 (PMC12402338; doi:10.1038/s42003-025-08683-8)
Supplement: Supplementary file 1 — Supplementary Information [file 42003_2025_8683_MOESM1_ESM.pdf]

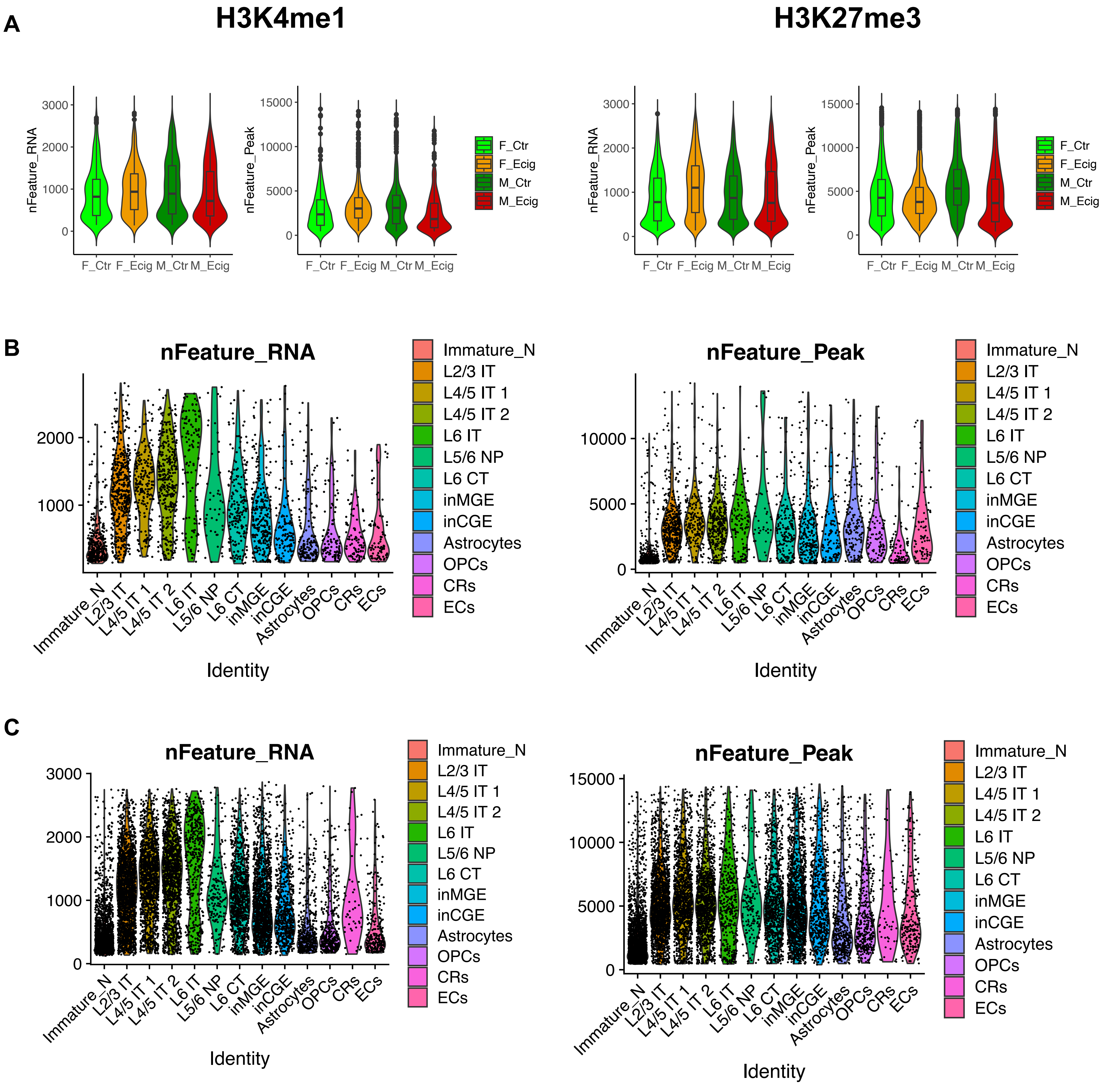

Supplementary Figure 1. QC metrics of the integrated H3K4me1 and H3K27me3 datasets

(**A**) Violin plots showing the number of unique genes (RNA feature) or unique peaks per nucleus in the 4 groups of the 2 histone mark Paired-Tag datasets, respectively. The middle line denoting the median value, whiskers show the maximum 1.5 interquartile range (IQR), and outliers were indicated with dots. In each group, nuclei were isolated from a pool of 4 rat brain PFC. Violin plots showing the numbers of unique peaks and unique genes (RNA feature) per nucleus at cluster level in H3K4me1 (**B**) and H3K27me3 (**C**) dataset. nFeature_RNA: the number of genes detected in each nucleus; nFeature_Peak: the number of unique peaks detected in each nucleus

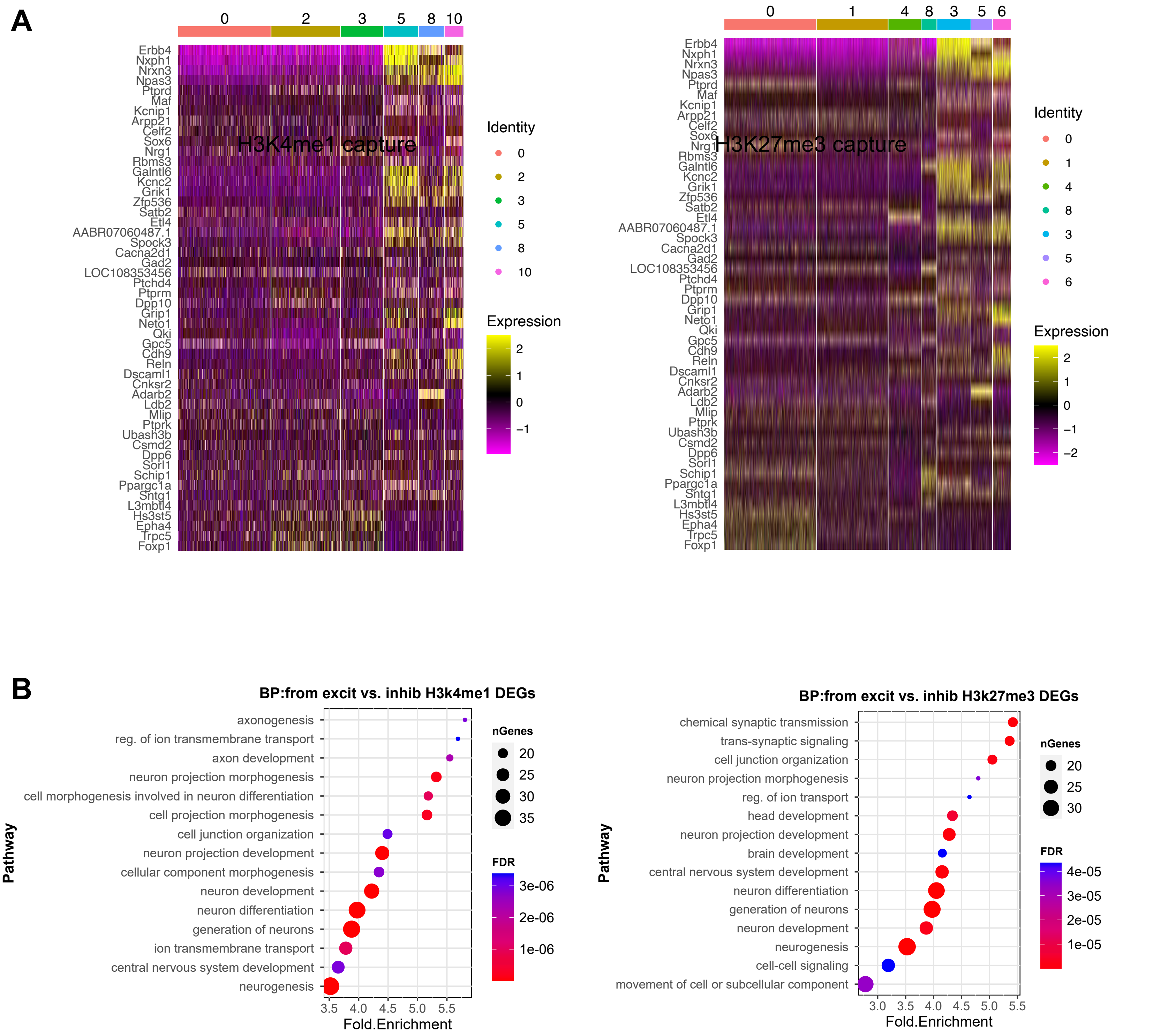

Supplementary Figure 2. Characterization of excitatory and inhibitory neurons

(**A**) Heatmaps showing the expression of cluster marker of excitatory neurons from H3K4me1 and H3K27me3 dataset, respectively. Only the clusters identified as excitatory neurons are included in the heatmap. (**B**) Gene Ontology Biological Processes enriched from differentially expressed genes between excitatory and inhibitory neurons. BP: Gene Ontology Biological Process.

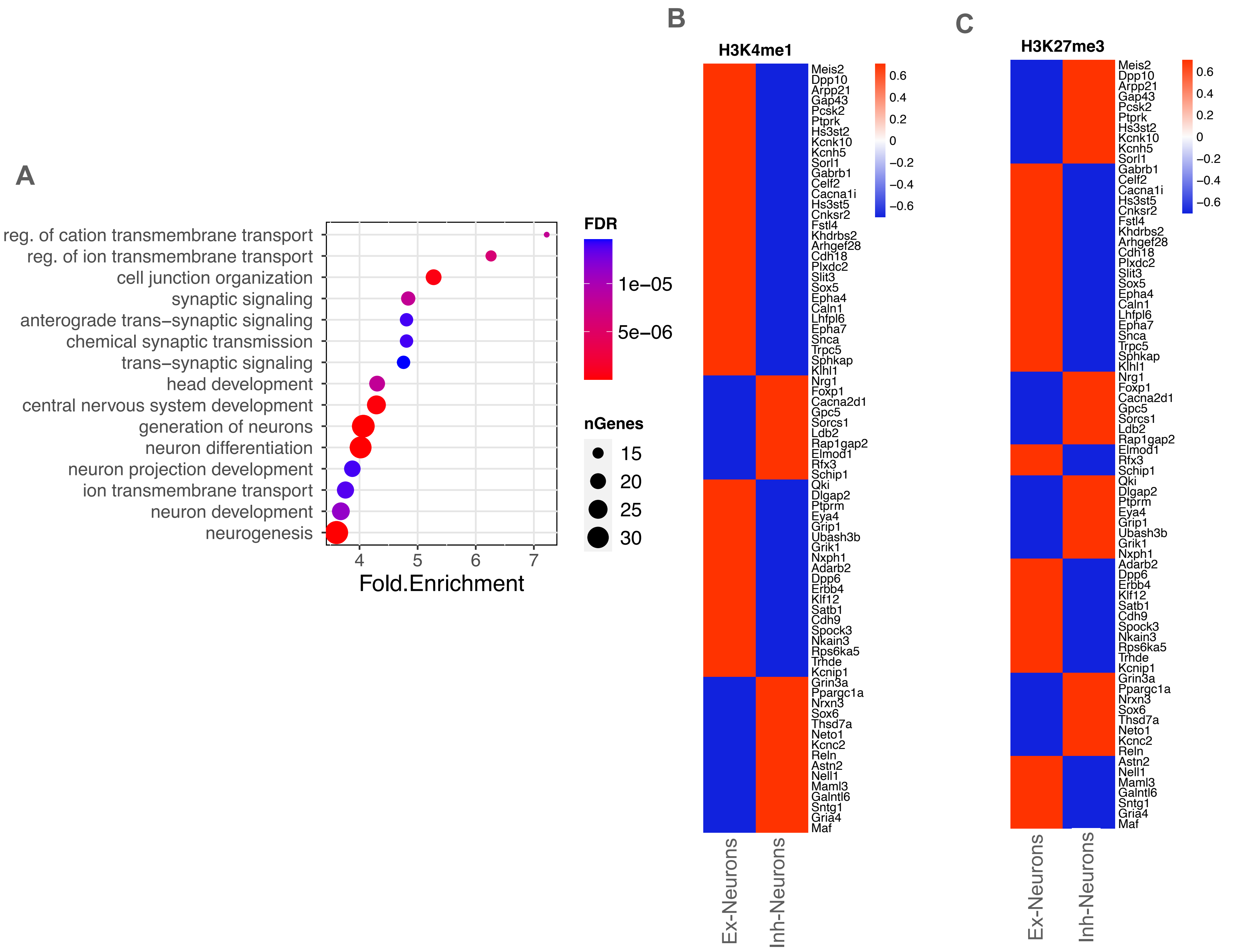

Supplementary Figure 3. Excitatory neuron specific DEGs and histone modifications

A) Gene Ontology Biological Process enrichment from excitatory neuron DEGs induced by prenatal e-cigarette aerosol exposure. H3K4me1 (**B)** and H3K27me3 (**C**) signal intensity at the promoter regions of the common DEGs identified in these two datasets.

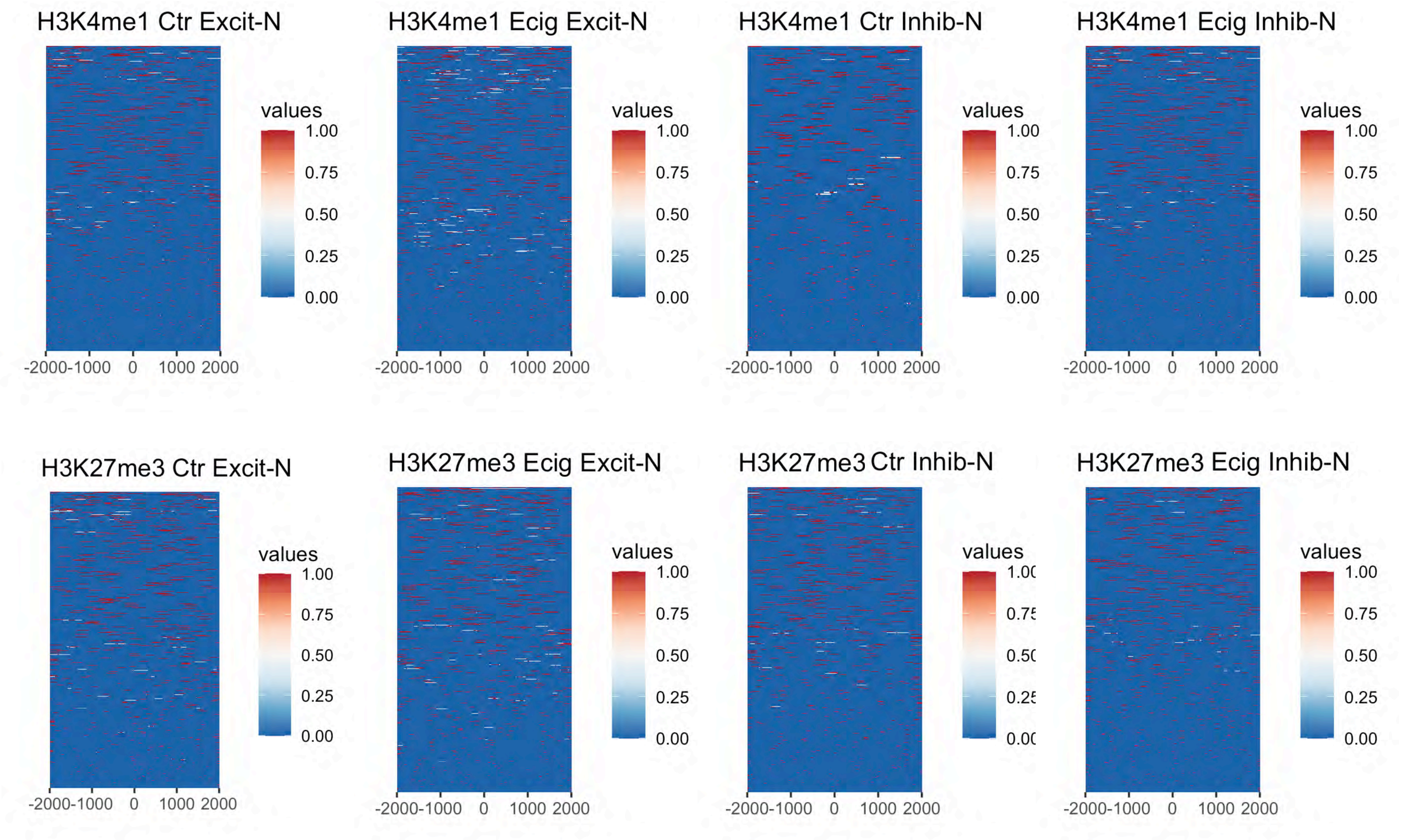

Supplementary Figure 4. Histone mark profiles around TSS in neurons

Each dash line represents a gene and only genes with histone mark signal at promoter (TSS±1000bp) were plotted.
