# Supplementary material for: Single-cell profiling of H3K4me1-H3K27me3 revealed bivalent regulation of abnormal neuronal development caused by prenatal e-cigarette vaporing
Source: Commun Biol. 2025 Sep 1;8:1326. doi: 10.1038/s42003-025-08683-8 (PMC12402338; doi:10.1038/s42003-025-08683-8)
Supplement: Supplementary file 2 — Description of Additional Supplementary Files [file 42003_2025_8683_MOESM2_ESM.pdf]

Description of Additional Supplementary files

File name: Supplementary Data 1

Description: Clusters and cell types from the two Paired-tag datasets.

File name: Supplementary Data 2

Description: Differentially expressed genes between excitatory neurons and inhibitory neurons.

File name: Supplementary Data 3

Description: Differential H3K4me1 and H3K27me3 peaks between excitatory neurons and inhibitory neurons.

File name: Supplementary Data 4

Description: Differentially expressed gene induced by prenatal e-cigarette aerosol exposure in each cell type.

File name: Supplementary Data 5

Description: Differential H3K4me1 peaks induced by prenatal e-cigarette exposure.

File name: Supplementary Data 6

Description: Differential H3K27me3 peaks induced by prenatal e-cigarette exposure.

File name: Supplementary Data 7

Description: The source data behind the graphs in the paper
