# Supplementary material for: Single-cell profiling of H3K4me1-H3K27me3 revealed bivalent regulation of abnormal neuronal development caused by prenatal e-cigarette vaporing
Source: Commun Biol. 2025 Sep 1;8:1326. doi: 10.1038/s42003-025-08683-8 (PMC12402338; doi:10.1038/s42003-025-08683-8)
Supplement: Supplementary file 10 — Reporting Summary [file 42003_2025_8683_MOESM10_ESM.pdf]

nature portfolio

Corresponding author(s): Charles Wang

Last updated by author(s): 2025/07/08

Reporting Summary

Nature Portfolio wishes to improve the reproducibility of the work that we publish. This form provides structure for consistency and transparency in reporting. For further information on Nature Portfolio policies, see our Editorial Policies and the Editorial Policy Checklist.

Statistics

For all statistical analyses, confirm that the following items are present in the figure legend, table legend, main text, or Methods section.

n/a	Confirmed	
☐	☒	The exact sample size (*n*) for each experimental group/condition, given as a discrete number and unit of measurement
☐	☒	A statement on whether measurements were taken from distinct samples or whether the same sample was measured repeatedly
☐	☒	The statistical test(s) used AND whether they are one- or two-sided<br>*Only common tests should be described solely by name; describe more complex techniques in the Methods section.*
☒	☐	A description of all covariates tested
☒	☐	A description of any assumptions or corrections, such as tests of normality and adjustment for multiple comparisons
☒	☐	A full description of the statistical parameters including central tendency (e.g. means) or other basic estimates (e.g. regression coefficient) AND variation (e.g. standard deviation) or associated estimates of uncertainty (e.g. confidence intervals)
☒	☐	For null hypothesis testing, the test statistic (e.g. *F*, *t*, *r*) with confidence intervals, effect sizes, degrees of freedom and *P* value noted<br>*Give P values as exact values whenever suitable.*
☒	☐	For Bayesian analysis, information on the choice of priors and Markov chain Monte Carlo settings
☒	☐	For hierarchical and complex designs, identification of the appropriate level for tests and full reporting of outcomes
☐	☒	Estimates of effect sizes (e.g. Cohen's *d*, Pearson's *r*), indicating how they were calculated

Our web collection on statistics for biologists contains articles on many of the points above.

Software and code

Policy information about availability of computer code

Data collection	DNA and RNA sequencing data was performed on an Illumina NextSeq 2000 at Center for Genomics, Loma Linda University.
Data analysis	Raw data processing and mapping was performed by Epigenome Technologies, INC (San Diego, CA), following the pipeline:<br>https://github.com/cxzhu/Paired-Tag.<br>Softwares used included:<br>bowtie2 2.4.5<br>STAR 2.7.0f<br>trim_galore 0.6.7<br>samtools 1.17<br>R packages:basilisk 1.14.3, zellkonverter 1.12.1, Seurat 5.0.1, SeuratData 0.2.2.9001, harmony 1.2.3, Signac 1.14.0, hdf5r 1.3.11, ChIPseeker v1.38.0<br>R version 4.3.1

For manuscripts utilizing custom algorithms or software that are central to the research but not yet described in published literature, software must be made available to editors and reviewers. We strongly encourage code deposition in a community repository (e.g. GitHub). See the Nature Portfolio guidelines for submitting code & software for further information.

Data

Policy information about availability of data

All manuscripts must include a data availability statement. This statement should provide the following information, where applicable:

- Accession codes, unique identifiers, or web links for publicly available datasets
- A description of any restrictions on data availability
- For clinical datasets or third party data, please ensure that the statement adheres to our policy

The datasets generated and analyzed in current study are available in the GEO repository with the access number GSE280559 and the following URL: https://www.ncbi.nlm.nih.gov/geo/query/acc.cgi?acc=GSE280559.

Research involving human participants, their data, or biological material

Policy information about studies with human participants or human data. See also policy information about sex, gender (identity/presentation), and sexual orientation and race, ethnicity and racism.

Reporting on sex and gender	N/A
Reporting on race, ethnicity, or other socially relevant groupings	N/A
Population characteristics	N/A
Recruitment	N/A
Ethics oversight	N/A

Note that full information on the approval of the study protocol must also be provided in the manuscript.

Field-specific reporting

Please select the one below that is the best fit for your research. If you are not sure, read the appropriate sections before making your selection.

☒ Life sciences ☐ Behavioural & social sciences ☐ Ecological, evolutionary & environmental sciences

For a reference copy of the document with all sections, see nature.com/documents/nr-reporting-summary-flat.pdf

Life sciences study design

All studies must disclose on these points even when the disclosure is negative.

Sample size	The material is single cells isolated from pooled cortices of 4 animals in each group. Because the effect size and standard deviation of prenatal e-cig exposue on brain development are impossible to measure, we will use a method based on ANOVA calculations and the degree of freedom which must be between 10 and 20 to determine the sample size (Ilyas, eta al., 2017). The calculation is based on law of diminishing return and an equation, called as “resource equation”. E = Total number of animals - Total number of groups. In our experiment, we had 4 groups (female e-cigarette, female control, male e-cigarette, and male control) and 4 animals in each group. So, the E values will be 12, which is large enough to satisfy the required degree of freedom (>10). As each cell is treated as an individual data point in single cell data analysis, we will only perform differential analysis on clusters with more than 50 cells.
Data exclusions	No data was excluded.
Replication	N/A
Randomization	N/A
Blinding	N/A

Reporting for specific materials, systems and methods

We require information from authors about some types of materials, experimental systems and methods used in many studies. Here, indicate whether each material, system or method listed is relevant to your study. If you are not sure if a list item applies to your research, read the appropriate section before selecting a response.

Materials & experimental systems

n/a	Involved in the study
☐	☒ Antibodies
☒	☐ Eukaryotic cell lines
☒	☐ Palaeontology and archaeology
☐	☒ Animals and other organisms
☒	☐ Clinical data
☒	☐ Dual use research of concern
☒	☐ Plants

Methods

n/a	Involved in the study
☒	☐ ChIP-seq
☒	☐ Flow cytometry
☒	☐ MRI-based neuroimaging

Antibodies

Antibodies used

Antibodies against H3K4me1 and H3K27me3 were purchased from Abcam (Cambridge, UK) and EpiCyphter (Durham, NC), respectively.

Validation

The specificity of these antibodies have been validated by Epigenome Technologies, INC (San Diego, CA) in a preliminary experiment and other similar Paired-tag experiment.

Animals and other research organisms

Policy information about studies involving animals; ARRIVE guidelines recommended for reporting animal research, and Sex and Gender in Research

Laboratory animals

Pregnant Sprague-Dawley (SD) rats were purchased from Charles River Laboratories (Portage, MI). The dams were exposed to e-cigarette aerosol or control air for a total of 17 days from gestation day 4 to day 20. P7 neonatal rats from either prenatal e-cigarette aerosol exposed or control dams were selected for this experiment.

Wild animals

N/A

Reporting on sex

Two male and two female P7 neonatal rats from either prenatal e-cigarette aerosol exposed or control dams were selected for this experiment (4 pups per dam for a total of 16 pups from 4 dams). Gender is a compound factor designed in this experiment. All differential analysis was conducted on gender specific and gender blind way. The gender difference in response to e-cigarette aerosol exposure was identified by comparing the differential gene expression and the signal intensity of histone modification.

Field-collected samples

N/A

Ethics oversight

All animal experiments involving animal care, surgery, and sample preparation were approved by the Institutional Animal Care and Use Committee of Loma Linda University and followed the guidelines by the National Institutes of Health Guide for the Care and Use of Laboratory Animals. All efforts were made to minimize animal suffering, and the number of animals used.

Note that full information on the approval of the study protocol must also be provided in the manuscript.

Plants

Seed stocks

N/A

Novel plant genotypes

N/A

Authentication

N/A
